# Supplementary material for: Complementary information derived from CRISPR Cas9 mediated gene deletion and suppression
Source: Nat Commun. 2017 May 23;8:15403. doi: 10.1038/ncomms15403 (PMC5457492; doi:10.1038/ncomms15403)
Supplement: Supplementary Information — Supplementary Figures [file ncomms15403-s1.pdf]

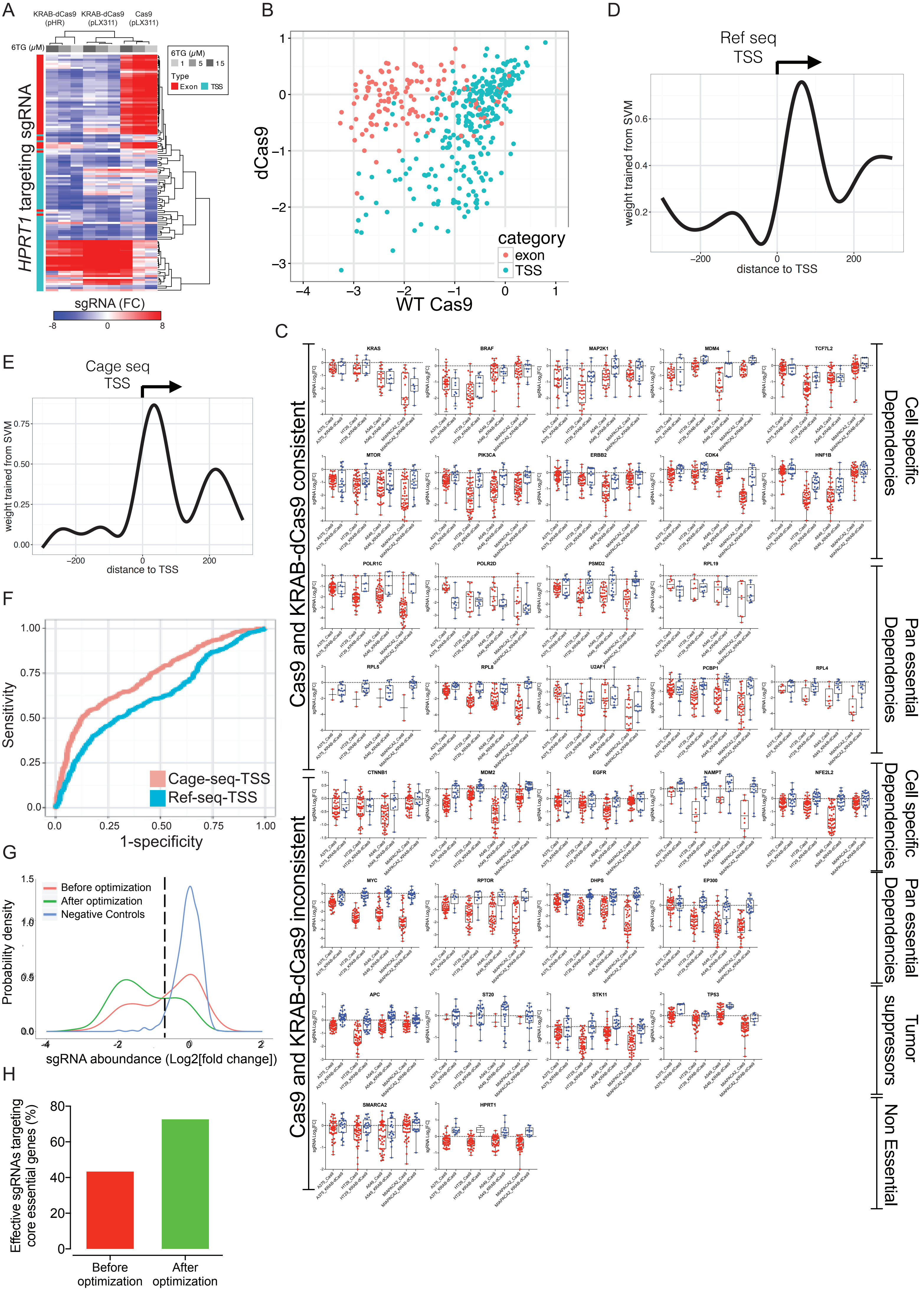

A
KRAB-dCas9 (pHR)
KRAB-dCas9 (pLX311)
Cas9 (pLX311)
6TG (μM)
1 5 15
Type
Exon TSS
HPRT1 targeting sgRNA
sgRNA (FC)
-8 0 8
B
dCas9
WT Cas9
category
exon
TSS
C
Cas9 and KRAB-dCas9 consistent
Cas9 and KRAB-dCas9 inconsistent
Cell specific Dependencies
Pan essential Dependencies
Tumor suppressors
Non Essential
KRAS
BRAF
MAP2K1
MDM4
TCF7L2
MTOR
PIK3CA
ERBB2
CDK4
HNF1B
POLR1C
POLR2D
PSMD2
RPL19
RPL5
RPL8
U2AF1
PCBP1
RPL4
CTNNB1
MDM2
EGFR
NAMPT
NFE2L2
MYC
RPTOR
DHPS
EP300
APC
ST20
STK11
TP53
SMARCA2
HPRT1
D
Ref seq TSS
weight trained from SVM
distance to TSS
E
Cage seq TSS
weight trained from SVM
distance to TSS
F
Sensitivity
1-specificity
Cage-seq-TSS
Ref-seq-TSS
G
Before optimization
After optimization
Negative Controls
Probability density
sgRNA aboundance (Log2[fold change])
H
Effective sgRNAs targeting core essential genes (%)
Before optimization
After optimization

Supplementary Figure 1: Loss of function screens with Cas9 and KRAB-dCas9 using a tiling sgRNA library. (A) Heat-map showing unsupervised hierarchal clustering of proliferation changes induced by suppression of *HPRT1* using different concentrations of 6TG and Cas9 or KRAB-dCas9 (in pLX311 or pHR). (B) Comparison of proliferation changes induced by Cas9 and KRAB-dCas9 following introduction of *HPRT1* targeting sgRNAs in A375 cells treated with 15 μM of 6TG. TSS targeting sgRNAs are colored in blue and exon targeting sgRNAs are colored in red. (C) Proliferation changes induced by sgRNAs targeting the indicated genes, in cells expressing Cas9 (red) or KRAB-dCas9 (blue). Only efficient sgRNAs (as determined by the sgRNA predictive algorithm) are shown for KRAB-dCas9 and only exon targeting sgRNAs are shown for Cas9. (D) Performance of sgRNA predictive model on core cell essential genes using NCBI ref seq TSS annotations or (E) FAMTOM CAGE-Seq TSS annotations. (F) ROC curve of sgRNA predictive model using TSS annotations from RefSeq (blue) or CAGE-Seq (red). (G) Distribution of sgRNAs targeting core cell essential genes before and after using the sgRNA optimization algorithm. (H) Percent of effective sgRNAs targeting core cell essential genes before and after sgRNA optimization algorithm.

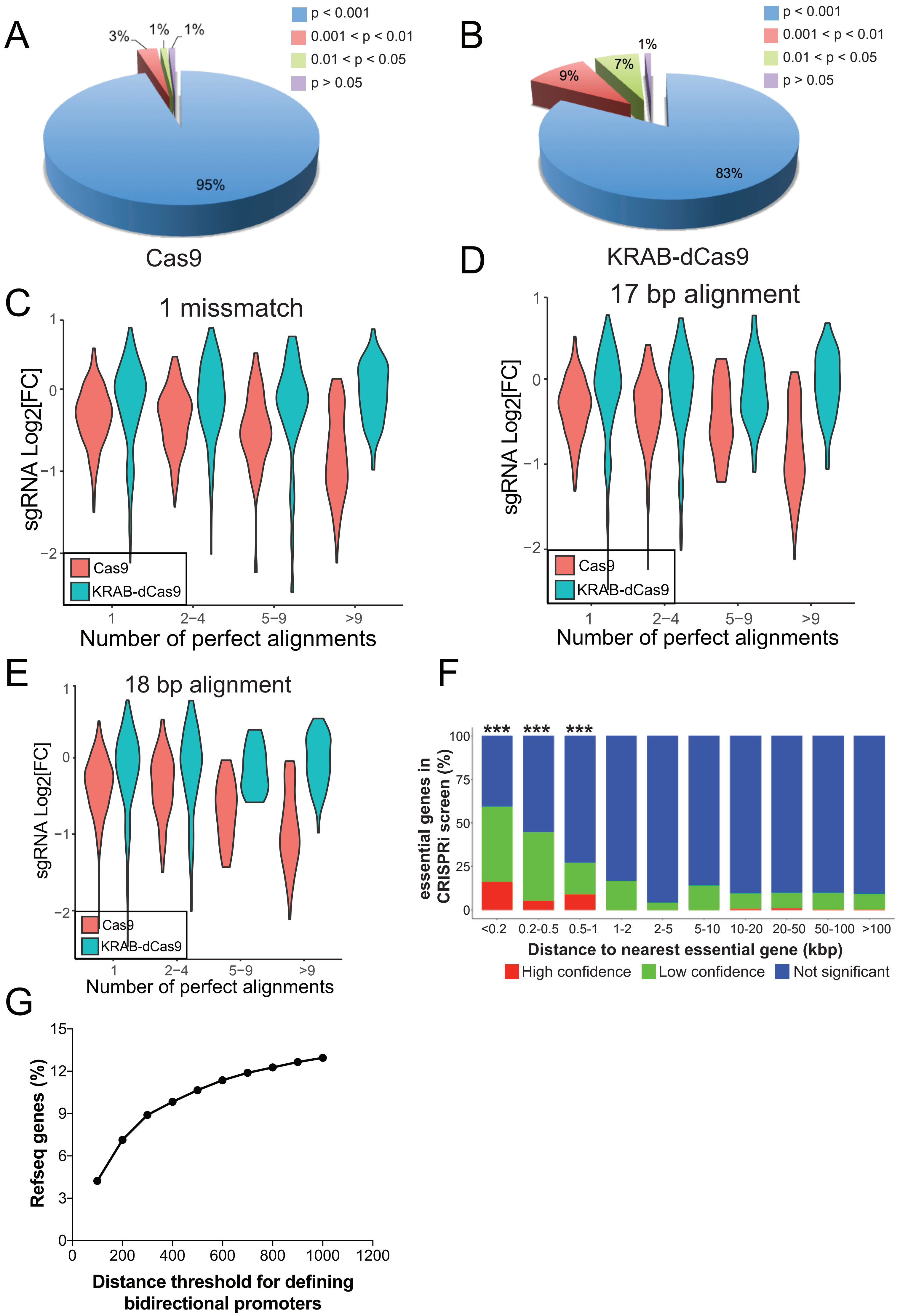

A
3%
1%
1%
95%
p < 0.001
0.001 < p < 0.01
0.01 < p < 0.05
p > 0.05
Cas9
B
9%
7%
1%
83%
p < 0.001
0.001 < p < 0.01
0.01 < p < 0.05
p > 0.05
KRAB-dCas9
C
1 missmatch
sgRNA Log2[FC]
Cas9
KRAB-dCas9
1
2−4
5−9
>9
Number of perfect alignments
D
17 bp alignment
sgRNA Log2[FC]
Cas9
KRAB-dCas9
1
2−4
5−9
>9
Number of perfect alignments
E
18 bp alignment
sgRNA Log2[FC]
Cas9
KRAB-dCas9
1
2−4
5−9
>9
Number of perfect alignments
F
***
***
***
essential genes in CRISPRi screen (%)
<0.2
0.2-0.5
0.5-1
1-2
2-5
5-10
10-20
20-50
50-100
>100
Distance to nearest essential gene (kbp)
High confidence
Low confidence
Not significant
G
Refseq genes (%)
Distance threshold for defining bidirectional promoters

Supplementary Figure 2: Comparison of Cas9 and KRAB-dCas9 experiments in identifying core essential genes. P-values were computed based on z-test, where the null distribution were computed based on sgRNAs targeting AAVS locus. (A) sgRNAs targeting exons were used for Cas9 analysis. (B) sgRNAs targeting primary CAGE-Seq TSSs were used in KRAB-dCas9 analysis. (C-E) Violin plot showing distribution of proliferation changes induced by intron-targeting sgRNAs that are aligned to multiple genomic loci, in Cas9 (red) or KRAB-dCAs9 (blue) expressing cells. (C) 1 mismatch was allowed when aligning these sgRNAs to the human genome. (D) Only 17 bp in 3' of the spacer are used for alignment. (E) Only 18 bp in 3' of the spacer are used for alignment. (F) Percent of genes that are scored to be cell essential in CRISPRi. Genes were categorized based on their distance to nearest cell essential gene scored in CRISPRc. (G) Percent of RefSeq genes that are associated with bidirectional promoters, which were defined based on the threshold of distance between the TSSs of two proximal genes transcribed towards opposite directions.
